# Concordance between clinical and radiographic evaluations of knee osteoarthritis

**DOI:** 10.1007/s40520-017-0847-z

**Published:** 2017-11-03

**Authors:** Camille Parsons, Nicholas R. Fuggle, Mark H. Edwards, Lyndsey Goulston, Anna E. Litwic, Darshan Jagannath, Suzan van der Pas, Cyrus Cooper, Elaine M. Dennison, The EPOSA Research Group

**Affiliations:** 1 MRC Lifecourse Epidemiology Unit, Southampton General Hospital, University of Southampton, Southampton, SO16 6YD UK; 2 Portsmouth Hospitals NHS Trust, Portsmouth, UK; 3 Department of Epidemiology and Biostatistics, EMGO Institute for Health and Care Research, VU University Medical Centre, Amsterdam, The Netherlands; 4 National Institute for Health Research Biomedical Research Centre, University of Southampton and University Hospital Southampton NHS Foundation Trust, Southampton, UK; 5 National Institute for Health Research Musculoskeletal Biomedical Research Unit, University of Oxford, Oxford, OX3 7LE UK

**Keywords:** Osteoarthritis, Patellofemoral, Tibiofemoral, Radiography, Epidemiology

## Abstract

**Background:**

Significant correlation has been previously demonstrated between radiographic and clinical diagnoses of knee osteoarthritis (OA); however, the specific findings on clinical examination that relate best to a radiographic diagnosis have not been fully elicited.

**Aims:**

We aimed to explore the relationship between clinical symptoms and physical findings with radiographic diagnoses of tibiofemoral and patellofemoral OA.

**Methods:**

This study was based on 409 individuals from the Hertfordshire Cohort Study, born between 1931 and 1939. Antero-posterior and lateral radiographs were taken of both knees. The presence of tibiofemoral and patellofemoral OA was defined according to the Kellgren and Lawrence score. Clinical symptoms, assessed using WOMAC, and physical findings were ascertained by examination. Relationships were assessed using multilevel univariate logistic regression.

**Results:**

In the 775 knees studied, the prevalence of physical findings was crepitus (25%), tibiofemoral tenderness (15%), bony swelling (12%), and pain on flexion (10%). Thirty-one percent (*n* = 238) knees demonstrated tibiofemoral OA, 28% (*n* = 220) showed patellofemoral OA, and 16% demonstrated OA in both locations. A global clinical symptom score was associated with increased risk of tibiofemoral OA (OR 12.5, 95% CI 5.4–29.0) and patellofemoral OA (OR 5.1, 95% CI 2.3–13.1). On clinical examination, the presence of crepitus, tibiofemoral tenderness, bony swelling, and pain on flexion was associated with increased risk of tibiofemoral OA; however, only tenderness was found to be associated with patellofemoral OA.

**Conclusion:**

Global clinical symptom score was associated with radiographic tibiofemoral and patellofemoral OA. However, individual clinical signs were more strongly associated with tibiofemoral than patellofemoral OA.

## Introduction

Osteoarthritis (OA) is the most common musculoskeletal condition in older adults. Nearly, 50% of those 75 years and above suffer from knee OA [1], which restricts mobility and causes stiffness, pain, joint swelling, and effusions. Many definitions of knee OA currently exist, with each definition having slightly different parameters and criteria for diagnosis. A clinical diagnosis of knee OA made using the American College of Rheumatology (ACR) criteria [2], European League Against Rheumatism (EULAR) [3], or National Institute for Health and Care Excellence (NICE) [4] guidelines focus on the presence of patient symptoms and clinical findings. However, radiographic knee OA definitions, such as Kellgren and Lawrence (K&L) [5], focus on structural changes within the joint.

The patellofemoral joint is associated with pain when walking up and down stairs [6, 7] and can be managed by conservative therapy such as quadriceps strengthening exercises, and rarely, surgical treatment such as patellofemoral arthroplasty. Epidemiological studies highlight that patellofemoral OA [8, 9] can occur in consort with tibiofemoral OA or in isolation, with some studies finding a significant difference in the demography of the two conditions [10]. There is a growing body of evidence to suggest that patellofemoral knee OA impacts independently on function and symptoms, and therefore, the aetiology and risk factors for tibiofemoral and patellofemoral knee OA may differ [7, 11].

It has been previously shown that there is modest agreement between a clinical diagnosis of knee OA, defined by the ACR criteria [2], and radiographic tibiofemoral OA, defined as a K&L score of greater than or equal to 2 [12–14]. Radiographic and clinical progressions of the disease are significantly associated, but the clinical relevance of the association is questionable [15]. Felson et al. investigated the relationship between symptoms of knee OA and radiographic findings. They assessed the correlation of clinical knee OA with a variety of definitions of radiographic knee OA and found reasonable levels of agreement between large osteophytes and the presence of clinical OA [16]. Furthermore, changes in symptoms and, more particularly, in structure over 3 years in patients with osteoarthritis have been shown to reflect a clinically relevant progression of the disease [17]. Previous studies have investigated clinical correlation of knee symptoms in patellofemoral disease using WOMAC [10, 18], reported symptoms [9] and pain [19], but these have not included physical, knee examination in their assessments. Other studies have focused on examining the prevalence of patellofemoral OA in specific ‘knee pain’ groups. These were recently meta-analysed and it was approximated that half the individuals with knee pain or radiographic OA had patellofemoral joint OA [20]. However, data regarding the prevalence in an unselected, elderly, western population, and the interaction between symptoms, clinical examination findings, and radiographic changes is lacking. Filling this void could enable physicians, particularly in the primary care setting, to predict which knee joint compartment is affected by OA using clinical examination. This would be useful as the treatments for tibiofemoral and patellofemoral OA may differ.

The relationship between clinical and radiological features of OA, relevant outcomes of the disease process, and how best to predict individuals at high risk of disease progression are important issues in this research area; clinicians considering who best to monitor for OA progression, and consider intervention, currently may adopt a clinical or radiological approach to defining who best to treat, and has been the focus of a number of recent papers and position statements [15, 21–25]. Indeed, the European Society on Clinical and Economic aspects of Osteoporosis and Osteoarthritis (ESCEO) considers that the major challenges in DMOAD development are the absence of a precise definition of the disease, particularly in the early stages, and the lack of consensus on how to detect structural changes and link them to clinically meaningful endpoints [22]. In related work, the ESCEO organised a working group to evaluate the need for updating the current European guidelines on clinical investigation of drugs used in the treatment of OA [23], and a recent position paper asked specifically whether we can identify patients with a high risk of OA progression who will respond to treatment? [24]. The “need for surgery” has been suggested as a potential relevant outcome, and was the subject of a recent paper that aimed to provide a contribution to the better definition of the “need for surgery” in advanced OA of the lower limbs [25].

This manuscript informs this debate; the aim of this study was to explore the associations between individual symptoms and physical findings in the knee, and radiographic tibiofemoral and patellofemoral knee OA among community dwelling older men and women who participated in the Hertfordshire Cohort Study (HCS) in the United Kingdom.

## Methods

### Study design

The study sample comprised of 207 men and 202 women from the HCS, and latterly, the UK component of the European Project on Osteoarthritis (EPOSA). The HCS is a large, prospective, population-based study of the life course origins of adult disease among community dwelling men and women born in Hertfordshire between 1931 and 1939 and still living in the county between 1998 and 2004 who were recruited because information on their early life was available. Both EPOSA and HCS studies have been previously described [26, 27], but the selection criteria for EPOSA were those individuals who were resident in Hertfordshire at the time of the study and who had a DXA scan and knee radiograph at two previous HCS study visits. A total of 592 HCS participants were eligible to participate in EPOSA, of whom 444 (75%) provided written, informed consent to participate in the study and of those 409 participants completed the study. Participants were visited at home by trained research nurses, who administered a questionnaire which included demographic information such as smoking status, alcohol intake, physical activity (recorded as minutes per day) and the Western Ontario and McMaster Universities Osteoarthritis Index (WOMAC)—a 24-item questionnaire with three subscales measuring pain, stiffness and physical function [28]. Each WOMAC response was scaled on a five-point Likert scale ranging from 0 to 4, with scores of 0 indicating none. WOMAC subscales were combined to provide global scores, with 1 or greater considered ‘symptom positive’ for the purposes of this study.

During the home visit, heights and weights of participants were obtained and a clinical examination of OA was conducted to assess the component parts required for a clinical diagnosis of knee OA (using ACR criteria) [2]. A trained nurse examined participants for signs of bone swelling (marked as present medially and laterally at the tibiofemoral joint line, with marginal bony swelling marked as absent) and joint tenderness, examined as follows: lateral tibiofemoral tenderness—with the knee flexed to 60°–90°, the examiner palpated the entire lateral tibiofemoral joint line from the lateral aspect of the patellar tendon to the lateral knee. Marginal tenderness was recorded as absent. On examination of medial tibiofemoral tenderness, the examiner palpated the entire medial tibiofemoral joint line from the medial aspect of the patellar tendon to the medial knee. Marginal tenderness was recorded as absent. Crepitus was defined by the presence of fine or coarse crepitations, using passive motion. Joint effusions were diagnosed if patellar tap or bulge sign was present.

Antero-posterior (AP) and lateral knee radiographs were taken of both knees and graded for OA by rheumatologists based on K&L scores [5]. Patellofemoral knee OA was defined as a patellofemoral K&L score of ≥ 2. Knees were excluded from the study if they had been previously surgically replaced.

The UK component of EPOSA had ethical approval from the Hertfordshire Research Ethics Committee, reference number 10/h0311/59, and all participants gave written, informed consent.

### Statistical analysis

Characteristics of study participants were described using means and standard deviations (SD) for continuous normal variables or median and inter-quartile range (IQR) for skewed continuous variables. Frequencies and percentages were used to summarise binary and categorical variables. Gender differences were analysed using the *t* test, Wilcoxon-Rank Sum test, and Chi-squared or Fisher’s exact tests as appropriate.

Knees were considered the unit of interest in statistical analysis, rather than study participant, and to enable multiply knees per study participants without biasing the results multilevel random intercept logistic regression was used. In this approach, radiographic knee OA was regarded as the ‘gold standard’ and was considered the outcome throughout. Statistical significance was defined at the 5% level and all analyses were undertaken using STATA 14 (StataCorp. 2015. Stata Statistical Software: Release 14. College Station, TX: StataCorp LP).

## Results

A total of 409 study participants and 775 knees were included within the study. Characteristics of these study participants are presented in Table 1, and the mean age at EPOSA baseline was just under 76 years in both men and women. On average, men were taller and heavier than women. Physical activity did not differ significantly between the sexes; however, on average, men consumed more alcohol than women and were more likely to be current or ex-smokers.

**Table 1:** Participant characteristics

	Men (*n* = 207), mean (SD)	Women (*n* = 202), mean (SD)	*p* value
Age (years)	75.5 (2.5)	75.7 (2.6)	0.425^a^
Height (cm)	172.8 (6.5)	158.8 (6.1)	< 0.001^a^
Weight (kg)	83.1 (12.0)	72.0 (13.6)	< 0.001^a^

	Median (IQR)	Median (IQR)	
Physical activity score (mins per day)	180.7 (105.0–267.9)	200.7 (135.0–280.0)	0.165^c^
Alcohol intake	6.3 (1.0–13.5)	0.4 (0.0–4.5)	< 0.001^c^

	*N* (%)	*N* (%)	
**Smoker status**			
Never	81 (39.1)	127 (62.9)	
Ex	115 (55.6)	69 (34.2)	
Current	11 (5.3)	6 (3.0)	< 0.001^b^
**Social class**			
I–IIINM	83 (42.1)	93 (46.0)	
IIIM–V	114 (57.9)	109 (54.0)	0.247^b^

^a^ *p* value for *t* test

^b^ *p* value for Chi square

^c^ *p* value for Wilcoxon-Rank Sum

**Fig. 1:**
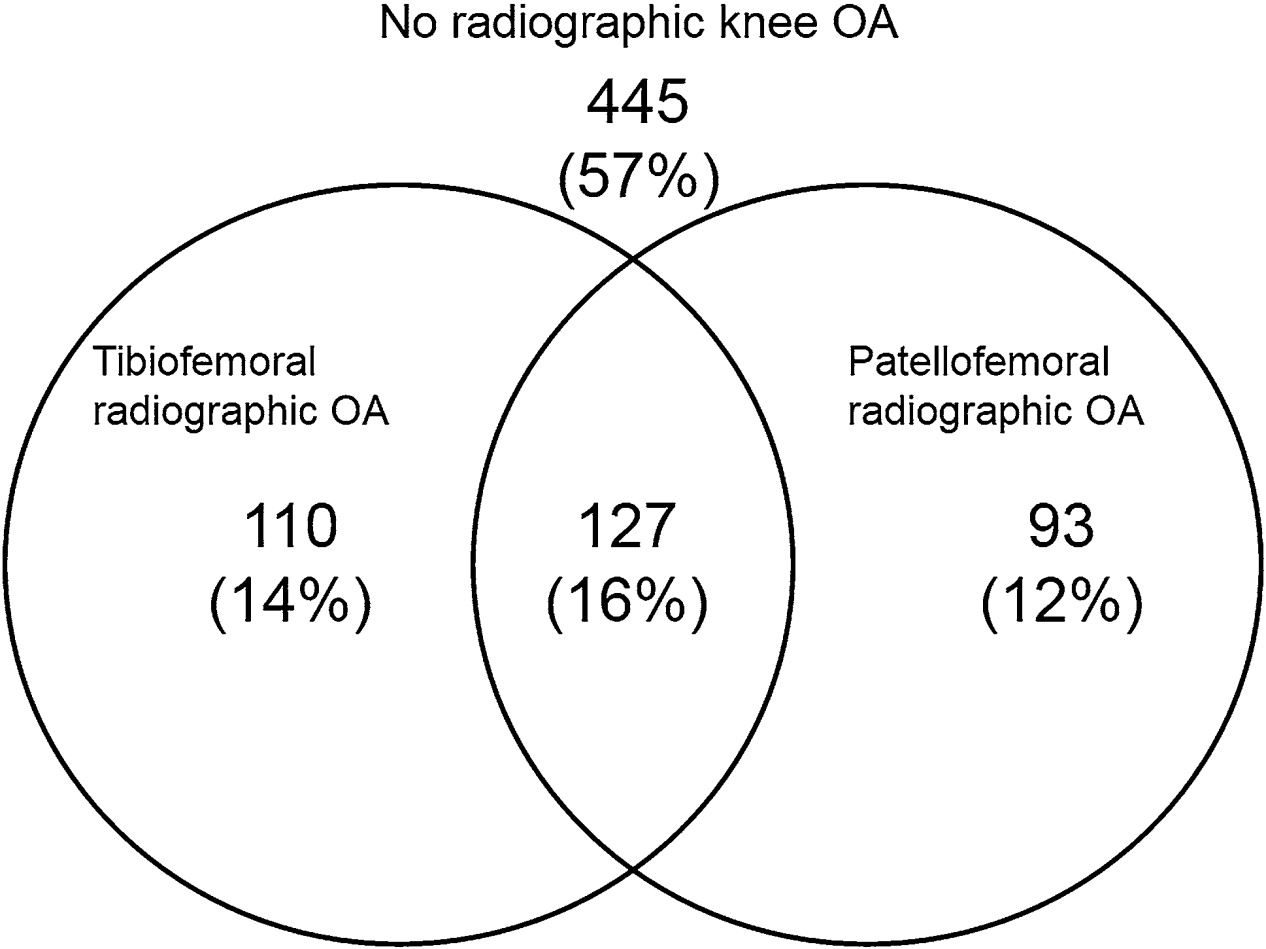
Venn diagram showing the overlap between tibiofemoral and patellofemoral radiographic knee OA

Table 2 presents the WOMAC symptoms of the study participants. Knee pain was reported by 34% of study participants and 31% reported experiencing knee stiffness. Nearly, 42% of study participants had some level of limited function and 52% had at least one of these WOMAC symptoms. Tibiofemoral radiographic knee OA was identified in 30.5% (*n* = 238) of the 775 knees and 28.4% (*n* = 220) were identified as having patellofemoral knee OA. As shown in Fig. 1, of those with tibiofemoral knee OA, 127 were also diagnosed with patellofemoral knee OA. When analysing all knees within the study, around one in six (16%) had both tibiofemoral and patellofemoral radiographic knee OA. Table 3 presents the physical findings within all knees. Crepitus was present in 24.8% of all knees, and was the most common physical finding. Tibiofemoral tenderness was present in 16.0% of all knees; bony swelling was present in 12.3% and pain on flexion present in approximately 10%. The least common physical finding in all knees was joint effusion, observed in 3.7% of knees. Crepitus was also the most common physical finding in those with tibiofemoral, patellofemoral, or both tibiofemoral and patellofemoral radiographic knee OA, affecting 35.1, 30.8, and 35.5% respectively. Tibiofemoral tenderness, bony swelling, and pain on flexion were more common in those with tibiofemoral than patellofemoral OA. However, joint effusion was more common in those with patellofemoral than tibiofemoral OA.

**Table 2:** Rates of WOMAC symptoms within study participants

Symptom	*N* (%)
Knee pain	139 (34.0)
Knee stiffness	127 (31.1)
Limited function	170 (41.6)
Global (combined)	213 (52.1)

**Table 3:** Rates of physical findings and disease severity within all knees

	All, *n* = 775 knees	Tibiofemoral OA, *n* = 238	Patellofemoral OA, *n* = 220	Tibiofemoral and patellofemoral OA, *n* = 127
	*n* (%)	*n* (%)	*n* (%)	*n* (%)
Clinical signs				
Crepitus	189 (24.8)	80 (35.1)	66 (30.0)	43 (35.5)
Tibiofemoral tenderness	123 (16.0)	61 (26.5)	50 (22.7)	35 (28.7)
Bony swelling	94 (12.3)	46 (20)	31 (14.1)	26 (21.3)
Pain on flexion	72 (9.5)	51 (18.1)	27 (12.3)	21 (17.5)
Joint effusion	28 (3.7)	17 (7.7)	19 (8.6)	16 (13.6)
Disease severity				
K&L tibiofemoral score				
0	253 (32.5)	–	29 (13.2)	–
1	287 (36.9)	–	64 (29.1)	–
2	197 (25.3)	197 (82.7)	106 (48.2)	106 (83.4)
3	37 (4.8)	37 (15.5)	19 (8.6)	19 (15.0)
4	4 (0.5)	4 (1.7)	2 (0.9)	2 (1.6)
K&L patellofemoral score				
0	183 (23.5)	16 (6.7)	–	–
1	374 (48.0)	95 (39.9)	–	–
2	218 (28.2)	125 (52.5)	218 (99.1)	125 (98.4)
3	2 (0.3)	2 (0.8)	2 (0.9)	2 (1.6)
4	0	0	0	0

**Fig. 2:**
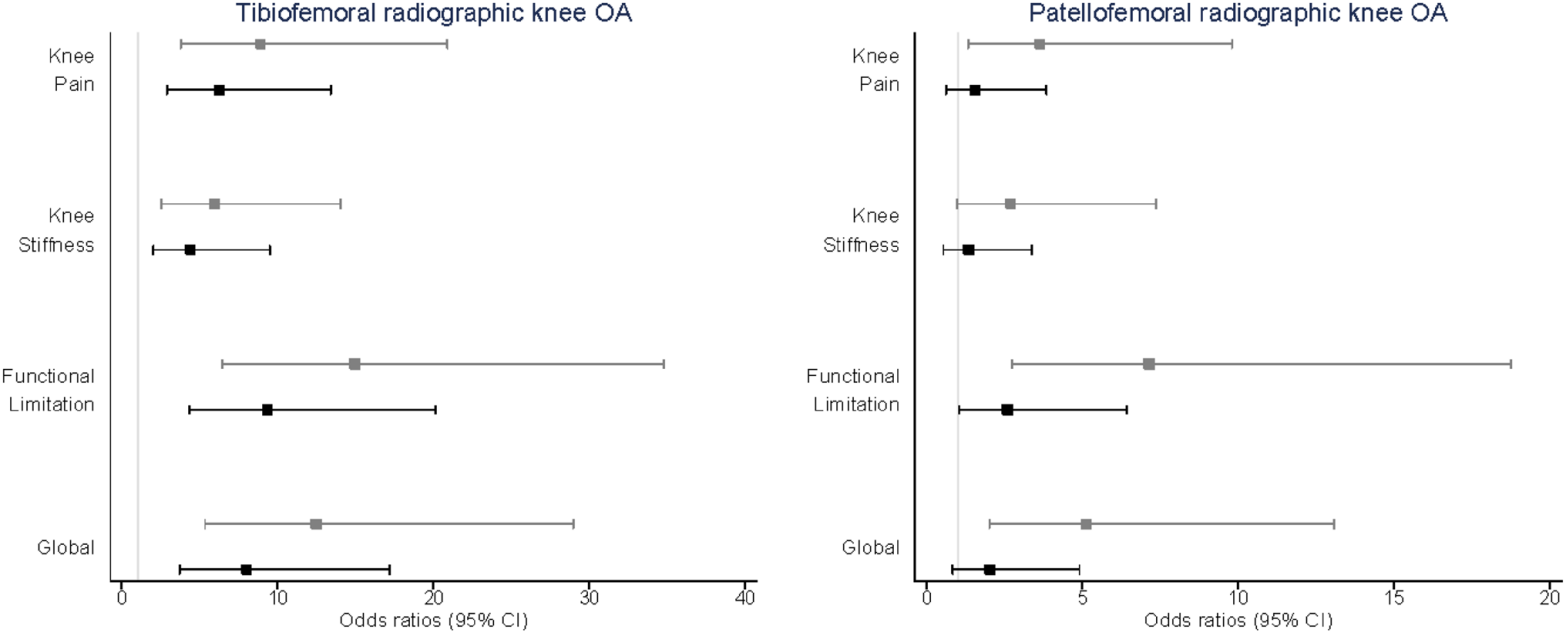
Relationship between WOMAC knee symptoms and radiographic knee OA

Figure 2 plots the odd ratios (OR) and 95% confidence intervals (CI) assessing the relationship between having tibiofemoral and patellofemoral radiographic OA in study participants with WOMAC knee symptoms; univariate associations are represented by the grey lines in the figure and mutually adjusted results are represented in the figure by the black lines. All symptoms were associated with a positive increase in the odds of having radiographic knee OA. Those experiencing knee pain had an OR of 8.9 (95% CI 3.8–20.9), those reporting stiffness had an OR of 5.9 (95% CI 2.5–14.0), and those with functional limitation had an OR of 14.9 (95% CI 6.4–34.8) of having tibiofemoral knee OA. The OR when assessing the relationship between tibiofemoral knee OA and any of these features globally was 12.5 (95% CI 5.4–29.0). When these associations were adjusted for the presence of patellofemoral OA, all relationships were attenuated, however, all remained statistically significant. Patellofemoral radiographic knee OA was not found to be associated with knee stiffness [OR 2.6 (95% CI 1.0–7.4)]. Knee pain [OR 3.6 (95% CI 1.4–9.8)], functional limitation [OR 7.1 (95% CI 2.7–18.7)], and a combination of any of these features globally [OR 5.1 (95% CI 2.0–13.1)] were all found to be positively significantly associated with patellofemoral knee OA. However, after mutual adjustment for tibiofemoral knee OA, these associations were almost all lost with only knee functional limitation remaining robust to adjustment [OR 2.6 (95% CI 1.0–6.4)].

**Fig. 3:**
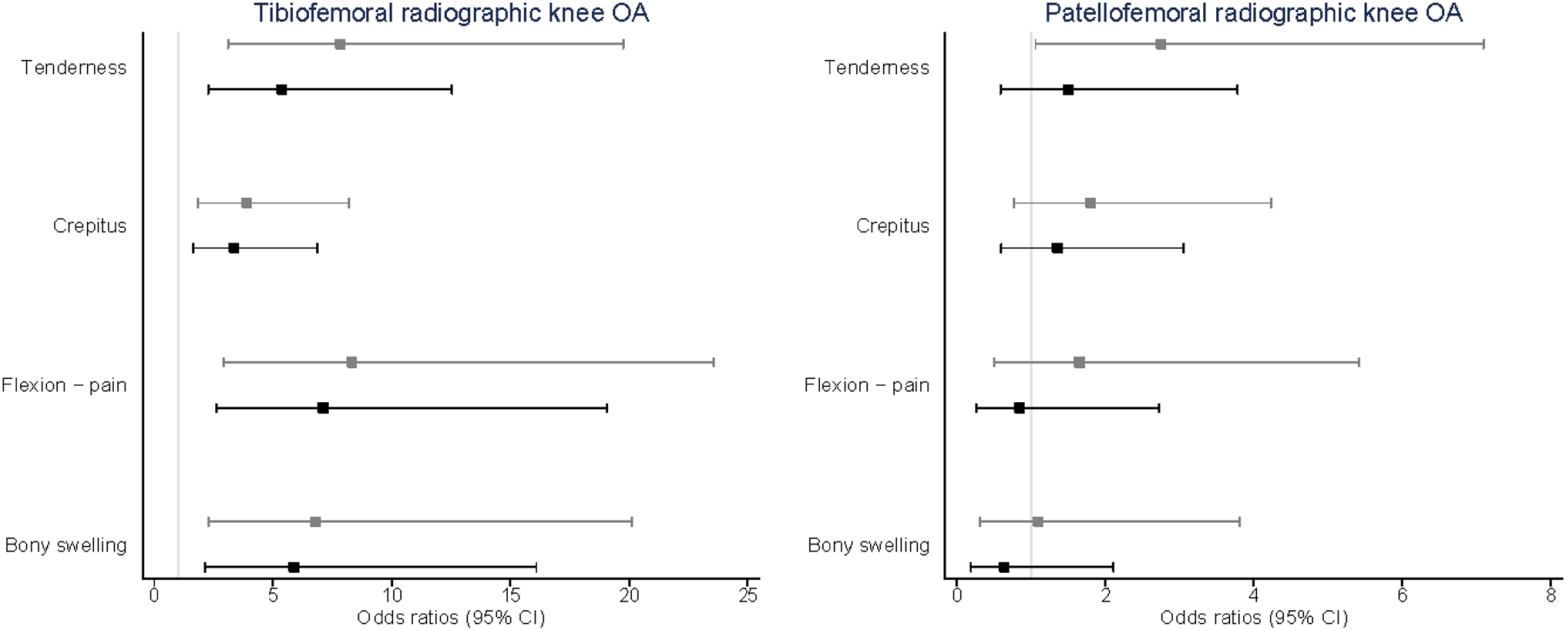
Relationship between physical findings and radiographic knee OA

The associations between the odds of having tibiofemoral and patellofemoral knee OA and physical findings are presented in Fig. 3, with the grey lines in the figure representing the univariate results and the black lines representing the mutually adjusted results. The presence of tenderness [OR 7.8 (95% CI 3.1–19.8)], crepitus [OR 3.9 (95% CI 1.8–8.2)], pain on flexion [OR 8.3 (95% CI 2.9–23.6)], and bony swelling [OR 6.8 (95% CI 2.3–20.1)] were all associated with increased odds of tibiofemoral knee OA. After mutual adjustment for patellofemoral knee OA, there was mild attenuation in the OR; however, the presence of all these physical findings remained associated with increased odds of having tibiofemoral knee OA. A weak association was found between the presence of tenderness and having patellofemoral knee OA [OR 2.7 (95% CI 1.1–7.1)]. This relationship did not remain after mutual adjustment for tibiofemoral OA. No significant association was found between the presence of pain on flexion, crepitus or bony swelling and patellofemoral knee OA. Due to the relatively small number of joint effusion, it was not possible to present a stable multilevel model, and consequently, the results of these analyses are not presented. Further adjustment for gender did not materially affect the results, although the relationship between functional limitation and patellofemoral OA was attenuated.

In those with tibiofemoral OA, 96 (40.3%) reported yes to pain going up and down stairs over the last week (question determined from WOMAC), 76 (34.2%) of those with patellofemoral OA had pain, and 59 (46.5%) of those with tibiofemoral and patellofemoral OA had knee pain when using stairs. Those who reported pain on using stairs had a significantly increased risk of tibiofemoral OA [OR 6.23 (95% CI 2.75–14.27), *p* value < 0.001], and also patellofemoral OA [OR 2.35 (95% CI 0.87–6.30), *p* value = 0.091], although this latter association did not reach statistical significance, suggesting that both forms of OA may be associated with pain on stair climbing.

Many individuals had coexisting patellofemoral and tibiofemoral OA; 110 subjects had tibiofemoral disease alone; 93 had patellofemoral disease alone and 127 had both tibiofemoral and patellofemoral disease. Although our primary approach to retain power was to adjust for disease at the other site, we also repeated analyses to consider associations in groups of individuals with only patellofemoral disease; only tibiofemoral disease; and combined disease. This second approach did not substantially change our results.

## Discussion

The aim of this study was to explore the associations between radiographic tibiofemoral and patellofemoral knee OA with individual knee symptoms and physical findings in the knee. A positive association was found between study participants with knee pain, knee stiffness, functional limitation, or any of these features globally, and tibiofemoral radiographic knee OA in univariate analysis. Similarly, the presence of tenderness, crepitus, pain on flexion, and bony swelling were all found to be positively associated with having tibiofemoral OA. When these associations were adjusted for the presence of patellofemoral knee OA, all relationships were attenuated but remained robust. The odds of having a K&L grade of 2 or more in the patellofemoral joint was found to be higher in study participants with knee pain, functional limitation, and any of these features globally in univariate analysis. No relationship was found between patellofemoral OA and knee stiffness and relationships were generally attenuated by adjustment for tibiofemoral OA. In addition, we found that pain on stair climbing was associated with a six-fold increased risk of tibiofemoral OA and a two-fold increased risk of patellofemoral OA.

Previous studies have shown correlation between WOMAC scores, K&L grades [29, 30] and ultrasound evidence of osteophytosis and cartilage thickness [29]. Interestingly, it has also been shown that ultrasound evidence of the location of OA within the knee can have an effect on symptoms. Pain on walking is more associated with lateral and medial tibiofemoral OA, and pain when climbing stairs has been found to be associated with both tibiofemoral and patellofemoral OA using both ultrasound and plain radiography [31]. This concurs with the clinical assertion that patellofemoral joint disease is associated with pain, particularly on ascending and descending stairs [6, 7] and supports our findings of a difference in the clinical manifestations of patellofemoral OA compared to tibiofemoral OA.

The burden of patellofemoral OA is evident in that about half the patients with radiographic knee OA or knee pain have patellofemoral disease [20]. The specification of knee pain as an inclusion criterion for studies of patellofemoral OA is common [11, 32], and so our study, which does not specify this, provides a prevalence of patellofemoral OA in the knee radiographs showed in this general, elderly population (28.4%). Indeed, timecourse studies have shown that the prevalence of patellofemoral OA increases over time and, often precedes the appearance of tibiofemoral disease [33], though such studies have investigated populations with a mean age in the 50s [32, 33]. Though Lankhorst and colleagues started with a lower proportion of patellofemoral arthritis (116 out of 845 participants), they found that from baseline to 5 year follow-up 66.3% of those with patellofemoral OA had developed tibiofemoral OA [33]. This is relevant for our study, which was carried out later in the life course, as increased age may explain the increased prevalence of patellofemoral disease. Prior studies have used WOMAC [10, 18], reported symptoms [9] and pain [19] in the context of OA of the compartments of the knee. We found a significant association between WOMAC knee symptoms (including pain on stair climbing), clinical signs and tibiofemoral OA, but no significant associations with patellofemoral OA following adjustment. This is interesting as previous studies have found an increased prevalence of knee pain [19], disability [9], and pain on ascending and descending stairs [34] in patellofemoral arthritis compared to tibiofemoral disease. This may be due to the relatively high prevalence of isolated patellofemoral OA at 12% compared to a recent study investigating patellofemoral OA in a cohort of older adults, Korean adults which found a 3.8% prevalence of isolated patellofemoral OA [10]. Similarly, to our findings, clinical scores including WOMAC and SF-36 did not significantly differ between isolated patellofemoral OA and non-OA groups (though they did not include clinical examination in their assessment). This could suggest that clinical symptom scores are less effective at eliciting patellofemoral OA in older adults, as the previously described studies [9, 19, 34] were performed in middle-aged demographics. Another potential reasons for the lack of association with WOMAC and clinical examination are the lack of examination for patellar tenderness. However, it should be noted that a recent, robust study of knee clinical examination concluded that no ‘typical’ examination findings were able to discriminate patellofemoral from tibiofemoral OA [35].

Cho and colleagues found that ‘traditional’ risk factors for knee OA including female sex, aging, and obesity were not associated with isolated patellofemoral OA [10]. They hypothesise that this may be due to a different aetiology and phenotype of patellofemoral disease. Further research in this area that disenables the influence of body composition on subtypes of OA may now advance, given the recent report that body composition assessed using bioelectrical impedance analysis might be associated with knee OA, and be a noninvasive tool for diagnosis of knee OA [36].

A more recent UK study sought to determine the clinical differences between medial and lateral patellofemoral joint OA finding that isolated lateral disease is more common, and more likely to be associated with the conventional predisposing factors for OA, than medial patellofemoral disease [18]. It is important to consider this within the context of our findings, as, in future studies, we may seek to investigate the differential signs and symptoms from medial and lateral patellofemoral OA.

Our study asks interesting questions with regard to the tools used for radiographical and symptomatic assessment. In terms of radiographic assessment, despite the World Health Organisation (WHO) adopting the use of K&L grades as the standard method of defining knee OA within epidemiological studies, it is not clear how the K&L grades should be applied to the patellofemoral joint separately from the tibiofemoral joint. Indeed, the assessments of radiographic features of patellofemoral disease are less reproducible than tibiofemoral features of OA [9]. In terms of symptom assessment, it should be noted that the WOMAC questionnaire is specific to the knee however is not specific for which knee is affected by OA, which makes our results more significant. It is interesting that the WOMAC parameters correlated with tibiofemoral OA but not with patellofemoral disease, perhaps suggesting a difference in the clinical phenotype of the two conditions.

This study was sizeable with the assessment of over 700 knees in older men and women, and extensive phenotyping of study participants. Although the HCS cohort, of which EPOSA is a subset, has been shown to be broadly comparable with the participants in the nationally representative Health Survey for England [27] a ‘healthy’ responder bias is unsurprisingly evident within the HCS [37], but is unlikely to have affected the inter-relationship between symptoms and physical findings in the knee and radiographic OA.

Our study includes some limitations. There may have been inconsistencies in the elucidation of clinical signs, although a recent study demonstrated “at least good” reliability for most clinical signs [38]. Patellar tenderness was not assessed, which has been associated with severe patellofemoral OA [11] and the WOMAC tool may have excluded those experiencing pain climbing up and down stairs, which, as previously noted [6, 7] is associated with patellofemoral OA. Inconsistencies may have occurred in the radiographic assessment of our participants; however, these will have been minimized by the use of two experienced rheumatologists to grade the radiographs, with good inter-observer agreement. Physical knee examinations for each study participant were performed by one of five specialist-trained nurses, to ensure data quality IOVs were undertaken using five study participants and similar levels of agreement existed for all variables evaluated. We have also previously reported good levels of agreement between- and within-observer variation for the clinical and radiographic features used within this current study; in brief, all clinical features were graded with good–excellent repeatability by multiple observers (*k* = 0.5–0.9) [39]. The WOMAC score, though widely utilized, is vulnerable to limitations, and in future studies, the Knee Injury and Osteoarthritis Score (KOOS) could be used, to include knee symptoms due to pathologies other than OA.

Our findings suggest that, in this elderly, western population, the use of a symptom-based tool is effective at suggesting the presence of radiographic patellofemoral OA. This may act as a helpful guide to physicians, particularly in a primary care context.

In conclusion, clinical symptoms and physical findings of OA were more closely related to tibiofemoral radiographic knee OA than patellofemoral OA.
